# Delayed Upper-Airway Injury after Accidental Alkaline Ingestion

**DOI:** 10.1155/2014/503205

**Published:** 2014-06-09

**Authors:** Matthew F. Ryan, Mindy Fernandez, Karen Laauwe

**Affiliations:** Department of Emergency Medicine, University of Florida, 1329 SW 16th Street, P.O. Box 1000186, Gainesville, FL 32610-0186, USA

## Abstract

A 62-year-old man presented to the emergency department one week after accidentally drinking an alkaline cleaning agent stored in unlabeled bottle. The day of the incident the patient presented to an outside hospital where he was admitted for an upper endoscopy of the esophagus which was found to be negative for acute injury. An initial chest X-ray taken the day of the incident was also found to be normal. After discharge the patient continued to have a sore throat and marked dysphagia which caused him to vomit repeatedly. Moreover, the patient began to develop chest pain with associated shortness of breath. We present a case of delayed airway injury and tracheal thickening and associated chest pain after alkaline ingestion and we discuss herein the pathophysiology and management of alkaline ingestions.

## 1. Introduction

Alkaline ingestion is a particularly concerning presentation for the emergency medicine physician. Alkaline agents can be extremely caustic and thus cause significant tissue damage to both the digestive and pulmonary tracts. The physical and chemical properties of caustic agents play an important role in injury severity and management; the concentration and the pH of the agent, the quantity ingested, the viscosity of the material, the contact time on internal tissues, whether the material was aspirated, whether the agent was solid, foam, or an aqueous solution all are important considerations. For example, household bleach (pH ~12) rarely causes significant injury if ingested [1] whereas solid agents such as lye or cleaning agents can lead to significant and permanent tissue injury and even result in death [2]. A key to alkaline injury is the fact that it causes liquefactive tissue necrosis leading to dissolution of cellular components and saponification of fatty tissues resulting in a liquid-gel amalgamation of dissolved cells and connective tissue. Ingestion of acidic media results in immediate denaturing of proteins which limits proteolysis of cellular constituents and leads to localized eschar formation which limits further tissue damage [3].

Herein we describe a case in which a patient presented with delayed respiratory symptoms and continued upper gastrointestinal symptoms a week after initial ingestion. We hypothesize that the patient suffered from continued yet insidious tissue destruction resulting from his progressively worsening symptoms. For example, because alkaline agents are not limited by decreased proteolysis or eschar formation, it is conceivable that tissue damage can continue after the initial insult due to infiltration of the alkaline material deep within the tissues. This case illustrates the importance of ensuring continue patient monitoring for caustic ingestions and thus minimizing subsequent untoward outcomes.

## 2. Case Report

A 62-year-old man presented to the emergency department one week after accidentally drinking an alkaline air-conditioner coil-cleaning agent stored in unlabeled bottle. The day of the incident the patient presented to an outside hospital where he was admitted for an upper endoscopy of the esophagus as well as laryngoscopy which were both found to be negative for acute injury. An initial chest X-ray taken the day of the incident was also found to be normal. After several days of observation the patient was discharged with a prescription for a proton-pump inhibitor. However, the patient continued to have a sore throat and marked dysphagia, dysphonia, and a sore throat which caused him to vomit nearly every time he attempted to eat or drink. He reported 11-kilogram weight loss from his decreased oral intake. Even more concerning was that the patient began to develop progressively worsening epigastric pain and upper chest pain with associated shortness of breath. The patient denied fever, chills, weakness, wheezing, bloody stools, or lower gastrointestinal upset. The ingestion was not an attempt of self-harm as the patient incorrectly assumed the unlabeled bottle contained a popular sports drink which is also brightly colored like the coil cleaning agent. Note that most commercial coil cleaning agents contain up to 5% sodium or potassium hydroxide solutions (pH > 13).

The patient's past medical history was significant for diabetes, mild chronic obstructive lung disease, and hypertension for which he is on an insulin regiment and lisinopril for his hypertension. On presentation, the patients vital signs were as follows: blood pressure of 133/77 mmHg, pulse of 111, respiratory rate of 20, temperature of 36.7°C and oxygen saturation of 96% on room air. His physical exam was significant for pharyngeal and uvula erythema and edema without ulcers. His lung exam demonstrated slightly diminished breathe sounds bilaterally with no noted increased work of breathing. His abdomen including the epigastric region was soft without rebound or guarding. The remainder of his exam was normal.

The patient's basic metabolic panel revealed bicarbonate of 21 mmol/dL, urea nitrogen and creatinine of 24 mg/dL and 1.27 mg/dL, respectively, and serum glucose of 157 mg/dL. Serum sodium, potassium, chloride, and liver enzymes were within normal ranges. A complete blood count (CBC) revealed a white count of 13.1 × 10^3^/mm^3^ with a hematocrit of 53.7%. Other CBC parameters were normal as was a serum lactate.

**Figure 1:**
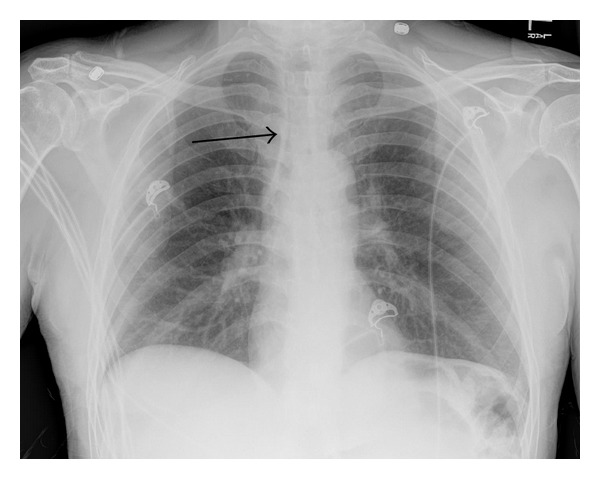
Anterior-posterior chest X-ray demonstrating tracheal wall thickening (arrow) as the result of alkaline ingestion.

An anterior chest X-ray (shown in Figure 1) was taken with showed clear lungs fields without effusion, infiltrate or aspiration, normal mediastinum, and no lymphadenopathy. The X-ray was significant for tracheal thickening not evident on the X-ray taken one week earlier. Subsequent upper endoscopy revealed mild diffuse esophageal erythema without no erosions or ulcers. The patient was discharged after two days with the recommendation for outpatient esophageal manometry and a pain management plan for his dysphagia. However, to date he continues to have dysphagia from permanent esophageal injury but no longer has any noted exacerbations of chest pain or dyspnea.

## 3. Discussion

Alkaline ingestion causes liquefactive corrosion to the tissues of the upper gastrointestinal tract which can lead to permanent laryngeal and esophageal injury and dysphagia [4] as was the case for the patient discussed herein. Liquefactive necrosis is of particular concern because if aspiration occurs (which likely occurred here given the patients significant tracheal thickening) upper respiratory tract injuries can result leading to dyspnea and chest pain. Moreover, cases of significant inhalation or aspiration can result in tracheobronchitis, lung parenchyma injury including hemopneumothorax and lung necrosis—which can be potentially life threatening—as well as airway bleeding and airway obstruction [5]. Aspiration may be more common than suspected due to vomiting and laryngeal edema from low viscosity alkaline agents as in this case. When acute airway compromise due to caustic ingestion and possible airway involvement is suspected, patients should be intubated early for airway protection [5]. Furthermore, the endotracheal tube itself serves as a stent to prevent obstruction from airway scarring and further tissue destruction. Airway establishment is best done under direct bronchoscopy to avoid iatrogenic trauma to the airway or placement of the endotracheal tube in a false passage.

Caustic injuries from alkaline injuries are particularly heinous because basic agents (pH > 7) can cause liquefaction of tissues forming a viscous gel of necrosed tissues primarily due to saponification of fatty substrates (e.g., cell membranes, neural sheaths, and intracellular vacuoles) and potentially result in full thickness burns. The extent of the tissue damage depends on the concentration of the offending agent, its viscosity, and the contact time of the agent. For example, concentrated crystalline materials such as powdered drain cleaners cause more extensive tissue damage than, say, household bleach which is a more dilute aqueous solution [6]. Alkaline coil cleaning agents have pH values up to 13.75 but by design have low viscosities to allow for permeation through the coils of cooling system. In our patient's case, the low viscosity likely prevented potentially life-threatening injuries, but the concentration of the ingested agent was enough to cause permanent sequelae in the way of dysphagia and epigastric and chest pain.

Management of caustic injuries includes bronchoscopy and esophagoscopy which are essential for initial diagnostic evaluation of the tissues of the upper gastrointestinal and pulmonary tracts and to gauge the extent of injury [4]. However, for alkaline injuries little can be done to abate the extent of the injury; gastric lavage, dilution of the agent, inducing emesis and activated charcoal, should be avoided. Steroids are still considered beneficial to avoid worsening tissue damage, stricture formation, and latent airway obstruction [7].

Most alkaline ingestions, as was the case here, are accidental. BB noted that 85% of all caustic ingestions in a retrospective analysis were accidental and patients with intentional ingestions had higher morbidity and mortality rates [6]. Nevertheless, the concern for self-harm should be part of patient management as needed to ensure patient safety.

## 4. Conclusion

Ingestion of alkaline agents is potentially life-threatening depending on the chemical and physical properties of the agent and of course the past medical history of the patient. Initial airway establishment may be necessary and vigilant monitoring for worsening conditions is vital to patient management. Admission for bronchoscopy and esophagoscopy to assess the extent of the injury is warranted in most cases. However, outpatient management is also vital to ensure patient safety. This may include pain management, repeated esophagoscopy with manometry, and medical management with proton-pump inhibitors and possibly steroids.
